# Microbiota Landscape of Gut System of Guppy Fish (*Poecilia reticulata*) Plays an Outstanding Role in Adaptation Mechanisms

**DOI:** 10.1155/2019/3590584

**Published:** 2019-02-17

**Authors:** Christian Aimé Kayath, Armel Ibala Zamba, Joseph Goma-Tchimbakala, Victor Mamonékéné, Gloire Moniceth Mombo Makanga, Aimé Augustin Lebonguy, Etienne Nguimbi

**Affiliations:** ^1^Institut National de Recherche en Sciences Exactes et Naturelles (IRSEN), Avenue de l'Auberge Gascogne, B.P 2400 Brazzaville, Congo; ^2^Laboratoire de Biologie Cellulaire et Moléculaire (BCM), Faculté des Sciences et Techniques, Université Marien Ngouabi, BP. 69 Brazzaville, Congo; ^3^Ecole Nationale Supérieure d'Agronomie et de Foresterie, Université Marien Ngouabi, BP. 69 Brazzaville, Congo

## Abstract

Microbial consortium that is present in fish gut systems works together to achieve unknown specific roles. Here, we collected guppy fish from hydrocarbon- and trace metal-contaminated wastewater to assess the relationships between gut microbiota and host fish adaptation. Targeted genes and 16S rRNA amplicon sequencing have been used to identify gut bacteria of guppies. Mineral-conditioned medium contributes to identify bacteria with the ability to grow and/or to tolerate hydrocarbon and trace metals. Additionally, trace metals' tolerance minimum inhibitory concentration (MIC) of microbiota was evaluated. We first isolated bacteria from the gut system, and we showed that *Bacillus* spp., *Staphylococcus* spp., *Shigella* spp., *Salmonella* spp, *Pseudomonas* spp., *Citrobacter* spp., *Salmonella enterica* ssp.*arizonae* sp., *Enterobacter* spp, and *Acinetobacter* spp. are part of guppy gut microbiota. Some representative species are able to degrade and/or tolerate gasoline and/or diesel fuel hydrocarbons. Tolerance to trace metals was observed in Gram-positive and Gram-negative bacteria. We showed that minimal inhibitory concentration (MIC) of some microbiota isolated from gut systems has been found including for mercury (Hg) between 2 and 4‰, cobalt (Co) Co (2 and 5‰), zinc (Zn) (9 and 18‰), and plomb (Pb) (22 and 27‰). Zn and Pb were the trace metals for which the rate of tolerance was significantly higher. Finally, we showed that cytochrome c oxidase is not interfering in presence of trace metals. The working consortium showed that bacteria should work together to achieve their best.

## 1. Introduction

Guppy fish has been introduced in many countries around the world. Its great adaptability including rapid reproduction and unquenchable appetite for mosquito larvae make it a valuable tool in combating a couple of diseases such as malaria that can be transmitted by mosquito bites [1]. In 1976, some collaboration between the Republic of Congo government and the World Health Organization (WHO) was made introducing *Poecilia reticulata* (Peters, 1959) in Brazzaville. One of the main approaches of this collaboration was to control mosquito epidemics for better fighting malaria. Guppies and mosquito larvae have been unfortunately coevoluated towards a balance relationship (Victor Mamonekene, data submitted). By the way, *P. reticulata* is a fascinating vertebrate fish model with a large landscape of facets linked to its ability to quickly grow by having a high population that is able to resist the environment pressures such as evolution of fish size [2], changes in predation environment and the ability to be anatomically modified [3], and behavioral trait in a population of individuals under the effect of natural selection [4]. This is creating a new phenotypic selection inducing a rapid escape ability [5]. Guppies' physiological changes linked to diets have involved gut length plasticity [6]. Fish guts contain microbiota playing different roles. Bacterial composition of the digestive tract of some fish has been documented. This depends on age, size, diet, and environmental conditions [7]. Different genera of lactic acid bacteria such as *Streptococcus*, *Leuconostoc*, *Lactobacillus*, *Lactococcus*, and *Carnobacterium* are also part of this microbiota [8]. Anaerobic bacteria such as *Escherichia coli* and *Streptococcus* sp. are the first to initiate colonization creating a propitious environment to the development of many other strict anaerobic microorganisms [9]. Numerous groups of microorganisms have also been successfully colonized guppy gut. Gut bacterial communities are now known to influence a wild range of fitness-related aspects of organisms. This includes Vibrionales, Bacillales, Actinomycetales, Clostridiales and Enterobacteriales [10]. Bacteria communities are known to play important physiological inputs by influencing metabolic processes, such as the digestion of complex carbohydrates [11, 12], regulation of fat storage and fish nutrition by microbiota [13], enzymes production from microbiota [14], and antibiotic resistance profile [15].

Most of the time, Brazzaville ecosystem waters contaminated with hydrocarbon are associated with trace metals. Some trace metals have been shown to be essential for a couple of biological functions for living organisms including Cu, Zn, Co, Fe, Mn, Ni, Cr, Se, and As, but the increase in their concentration can lead to phenomena of toxicity to organisms. Associations between trace metals and *P. reticulata* have been documented [16]. Some heavy-metal concentrations such as Cd, Cr, Cu, Hg, Pb, and Zn have been identified in the muscle tissue of some fish species including guppies [17–19].

Wastewater fish are exposed to high hydrocarbon concentrations and trace metals. Several investigations are still unclear in terms of adaptive mechanisms linked to the gut microbial communities. Adaptation strategies of guppies living in wastewaters contaminated with hydrocarbon and heavy metal involving gut microbial populations is still missing. This work aims to study the role of microbiota in the adaptive mechanisms in wastewaters. By isolating intestine bacteria of gut track and identifying microbiota using *icsB*, *invG*, and *16S rRNA* gene, by determining the emulsion index (E24), by investigating the capacity to grow and/or to tolerate in mineral media supplemented with hydrocarbons, by calculating minimal inhibitory concentration (MIC) of heavy-metal inhibition, and by studying interferences between cytochrome oxidases and trace metals, this study will allow contributing to the understanding of the knowledge gap. This work will try to provide a deep assessment of adaptation mechanisms occurring in the guppy gut system once in contact with pollutants.

## 2. Materials and Methods

### 2.1. Collection of Guppies and Isolation of Gut Microbiota

Fresh wastewater guppies were collected from different gutters close to the laboratory and from small streams of Brazzaville and stocked in ziplocks and transported from the site of sampling to the laboratory (lab GPS coordinates: elevation (127 m), distance (5.7 km), S04.27643°, E 015.29297°; station GPS coordinates: elevation (127 m), distance (6.1 km), S04.27643°, E 015.29297°). Fish had been collected by using deep net. Samples were systematically brought from the station to the laboratory (about 0.4 km) for dissection. Tricaine methane-sulfonate (MS-222) has been used as an anesthetic and euthanasia agent, and the fish was surface-sterilized with alcohol (70%). The intestine of *Poecilia reticulata* gut track was removed by dissection with sterile instruments and then washed in 70% ethanol to avoid contamination. The intestine was immersed in sterile saline. This was vigorously vortexed to separate microbiota from tissue. Dilutions were done, and bacterial suspension was streaked on nutrient agar media. Enumeration of colonies was done in triplicate on plate count agar (PCA). The Petri dishes were incubated at 37°C for 24 h to 48 h. After the first isolation on Petri dishes, different colonies were obtained. Each colony of different appearance was separately isolated. Purification of the isolates was rigorously done by successive and alternating subcultures. Purity was estimated by using a microscope for morphological characterization. Gram status was determined by using 3% KOH. Sporulation, hydrogen peroxide (H_2_O_2_), and oxidases tests were used for biochemical characterization.

### 2.2. Identification of Isolate and Genomic DNA Extraction and Sequencing

Conventional methods and Enterosystem 18R (Liofilchem kits) were first done for identification of all Gram-negative bacterial strains. This was performed according to the manufacturer's instructions. SS medium has been used for *Shigella* spp. and *Salmonella* spp. preidentification. To easily confirm *Shigella* or *Salmonella*, targeted primers were used. For *Shigella* spp., ACKicsBs (5′-ATGAGCCTCAAAATTAGCAA-3′) and ACKicsBas (5′-CTATATATTAGAATGAGAGTTATTC-3′) primers have been used by direct amplification from colonies. In terms of *Salmonella* spp., ACKinvGs primer (5′-ATGAAGACACATATTCTTTTGGCC-3′) and ACKinvGas primer (5′-TCATTTAATTGCCTCCTGACCTCTA-3′) have been used by the same method as *Shigella*. For other bacteria, genomic DNA extraction and purification was performed using NucleoSpin Microbial DNA kit (Macherey-NAGEL). Briefly, the targeted isolate is grown in 5 mL of LB broth for 24 h at 37°C with stirring. DNA purity was assessed by electrophoresis on 1% agarose gel and by the ratio of optical densities 260/280 nm. The housekeeping 16S rRNA gene has been amplified by PCR (Thermal Cycler, Bio-Rad) by using universal primers fD1 (5′-AGACTTTGATCCTGGCTCAG-3′ and rP2 (5′-ACGGCTACCTTGTTACGACTT-3′). 5 *μ*L of each amplification product was mixed with 2 *μ*L of loading buffer (BIOKÉ). Mixtures were subjected to electrophoresis on 1% agarose gel (w/v). The 10 kb 2-Log (BIOKÉ) was used as a molecular weight marker. The PCR products were purified using the solution of Gel Extraction kit (Omega Bio-tek), and the purified products were subjected to sequencing by the Sanger technique (3130 × l Genetic Analyser (Applied Biosystems)). The sequences obtained were aligned with the software BioNumerics 7.5 (Applied Maths, Belgium) and corrected manually to resolve discrepancies between the sense and antisense strands. Sequences were compared with homologous sequences contained in the sequence databanks through NCBI (National Center for Biotechnology Information (http://www.ncbi.gov/Blast.cgi) using the BLASTn program based on the identification criterion published by Drancourt [20]. All sequences have been stored in NCBI GenBank data.

#### 2.2.1. Tolerance of Microbiota to Hydrocarbon and Evaluation of Emulsion Index (E24)

The capacity of bacterial isolates and consortium to degrade hydrocarbons was randomly evaluated according to the capacity to utilize gasoline and diesel fuel hydrocarbons. An additional test was performed by studying the production of biosurfactants that emulsify hydrocarbons. The emulsion index (E24) was calculated as an indicator for biosurfactants production. McFarland standards were used as a reference to adjust the turbidity of bacterial consortium. Isolates and consortium were cultivated for 14 days at 37°C by using an adapted Bushnell-Haas (BH) mineral salt media composed of 10 g/L NaCl, 0.29 g/L KCl, 0.42 g/L MgSO_4_.7H_2_O, 0.83 g/L KH_2_PO_4_, 0.42 g/L NH_4_SO_4_, and 1.25 g/L K_2_HPO_4_ [21]. The medium was adjusted to pH 7.2 and supplemented with gasoline or diesel fuel (1 mL for 300 mL of medium). This experiment was done in triplicate. The E24 was investigated by adding crude oil with LB medium in 1 : 1 ratio (v/v). The solution was vortexed for 5 min and incubated for 24 h. The emulsion rate was calculated through the height of the emulsion layer. In addition, E24 was determined for gasoline and diesel fuel hydrocarbons. All the experiments were performed in triplicates, E24 = height of emulsion layer/total height of solution × 100.

### 2.3. Determination of Minimal Inhibitiory Concentration (MIC)

The isolates were subsequently submitted to their capacity to tolerate trace metals. Four different heavy metals combined with salt were used including PbNO_3,_ HgCl_2_, ZnCl_2_, and CoSO_4_. All the solutions were prepared in deionized water with specific concentrations. The buffer stock solution was diluted to the working concentration as required. Controls for overnight inoculum viability and density were performed in tryptic soy broth at 37°C. 100 *µ*L of randomly chosen strain cultures in accordance with McFarland standards was plated on LB Petri dishes for overnight subcultures. 50 *µ*L of metal working concentration was deposited on Petri dishes. Cultures were incubated at 37°C, and MICs were evaluated.

### 2.4. Effect of Metals Interference on Cytochrome c Oxidase Activity

The metal interferences on the production and expression of cytochrome c oxidase in metal-tolerant strains were assessed indirectly by the microbiological oxidase test. For this experiment, randomly chosen strains with positive results in the presence of the metals were analyzed. LB medium was used for culture supplemented with PbNO_3_ (15‰), HgCl_2_ (1.5‰), ZnCl_2_ (8‰), and CoSO4 (1.5‰). All tests were made in triplicates. The inoculation conditions, incubation, and reading of the tests were in accordance with the methods of McFarland.

### 2.5. Statistical Analysis

Principal component analysis (PCA) was used to investigate possible correlations between growth after 2 to 8 and 11 days or heavy trace metals and (1) strains assemblages or (2) strains assemblages and Consortium of Gram-negative/Gram-positive bacteria groups. Prior to ordination, strains abundance data were transformed to better meet the assumptions of normality [22] using ln (*x* + 1). In addition, a PCA of cytochrome oxidase enzymatic activity was conducted in order to see the effect of metals on enzymatic activity. All analyses were performed using CANOCO (Canonical Community Ordination, version 4.5) [23].

## 3. Results

### 3.1. Microbiological and Biochemical Assessment

**Figure 1:**
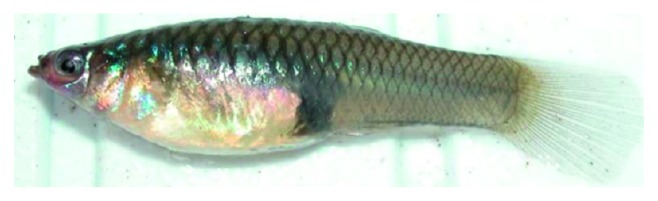
*Poecilia reticulata *collected from wastewaters. Female (37.5 mm SL).

In this study, the intestine of *Poecilia reticulata* (Figure 1) gut track has been aseptically dissected as mentioned in Methods and Materials.

Seventy-two (72) isolates have been purified and obtained (Table 1). The 3% KOH Gram test identified 25% of Gram-negative cultural bacteria and 75% of Gram-positive cultural bacteria. In order to estimate the total number of microbial florae contained in the digestive tract of guppy fish on PCA media, male and of female guppy fish have been dissected. Using PCA medium, we found 1.3 ± 0.51 (10^5^ UFC/ml/intestine track) in the male gut system and 1.5 ± 0.68 (10^5^ UFC/ml/intestine track) in the female gut system. Mossels, SS, Chapman media, and Enterobacter, and Enterosystem 18R allowed the presumptive identification. 66.66% (48) of Bacillaceae are the most dominant genera in the digestive tract of *P. reticulata* isolated from Brazzaville wastewater, 8.33 % (6) of Staphylococcaceae and 25 % (18) Enterobacteriaceae. The identification of *Shigella* spp. and *Salmonella* spp. has been confirmed using direct PCR of targeted specific genes such as *icsB* and *invG*, respectively. Using *16S rRNA* gene, *Acinetobacter haemolyticus* (GenBank: MK099885.1), *Bacillus* spp. including *Bacillus subtilis* (GenBank: MK099888.1), *Bacillus cereus* (GenBank: MK099886.1, MK099887.1, and MK099891.1), *Bacillus amyloliquefaciens* (GenBank: MK156314.1) and *Bacillus licheniformis, Bacillus altitudinis* (GenBank: MK099889.1 and MK156313.1) *and Bacillus megaterium* (GenBank: MK099890.1, MK391976.1, MK391968.1, MK391975.1, and MK391970.1), *Bacillus anthracis* (GenBank: MK391960.1) and *Bacillus marisflavi* (MK391969.1, MK391972.1, and MK391973.1) have been identified (Table 1).

### 3.2. Ability of Microbiota to Degrade and/or Tolerate Hydrocarbons

**Figure 2:**
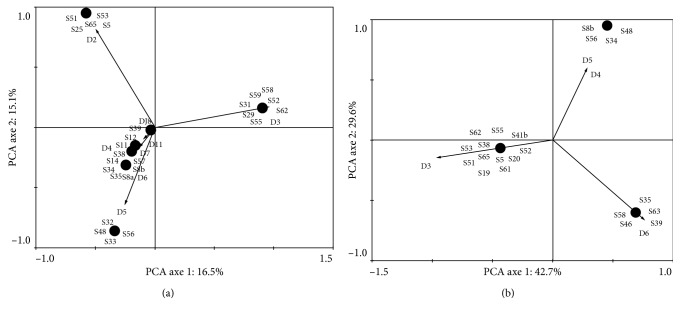
PCA of bacteria showing abilities to grow in Bushnell-Haas (BH) mineral salts medium composed of 10 g/L NaCl, 0.29 g/L KCl, 0.42 g/L MgSO4·7H_2_O, 0.83 g/L KH_2_PO_4_, 0.42 g/L NH_4_SO_4_, and 1.25 g/L K_2_HPO_4_ and supplemented with gasoline (a) and diesel fuel (b). D2, D3, D4, D5, D6, D7, D8, and D11: growth after 2, 3, 4, 5, 6, 7, 8, and 11 days.

To investigate the relationship between hydrocarbon and gut system bacteria, we first evaluate the ability of microbial strains to degrade and/or to tolerate hydrocarbons by using the BH medium supplemented with gasoline and diesel fuel hydrocarbons. The results after 14 days of incubation are shown in Figure 2. Among the 72 isolates obtained, basing on gasoline hydrocarbons criteria, 36.1% (26) of strains are able to degrade this type of fuel. S51, S25, S65, S5, and S53 are able to grow after 2 days. S31, S29, S55, S62, S58, S59, and S52 are able to grow after 3 days. S32, S48, S56, and S33 are able to grow after 5 days (Figure 2(a)).

For diesel fuel criteria, 29.1% (21) are able to degrade and/or to tolerate diesel fuel hydrocarbons. Most strains including S62, S55, S41b, S38, S65, S51, S5, S20, S19, and S61 grow after three days. S48 strain was able to grow after 5 days but S34, S46, and S8b were able to grow after 4 days. Some strains have been grown after 6 days including S35, S58, S63, S46, and S39 (Figure 2(b)). Together with both criteria, 20.83 % (15) are able to degrade gasoline and diesel fuel hydrocarbons. This includes S35, S34, S62, S52, S39, S65, S38, S5, S35, S55, S48, S62, S51, and S53 (Figures 2(a) and 2(b)).

### 3.3. Ability of Bacteria to Produce and to Secrete Biosurfactant in Extracellular Area

**Figure 3:**
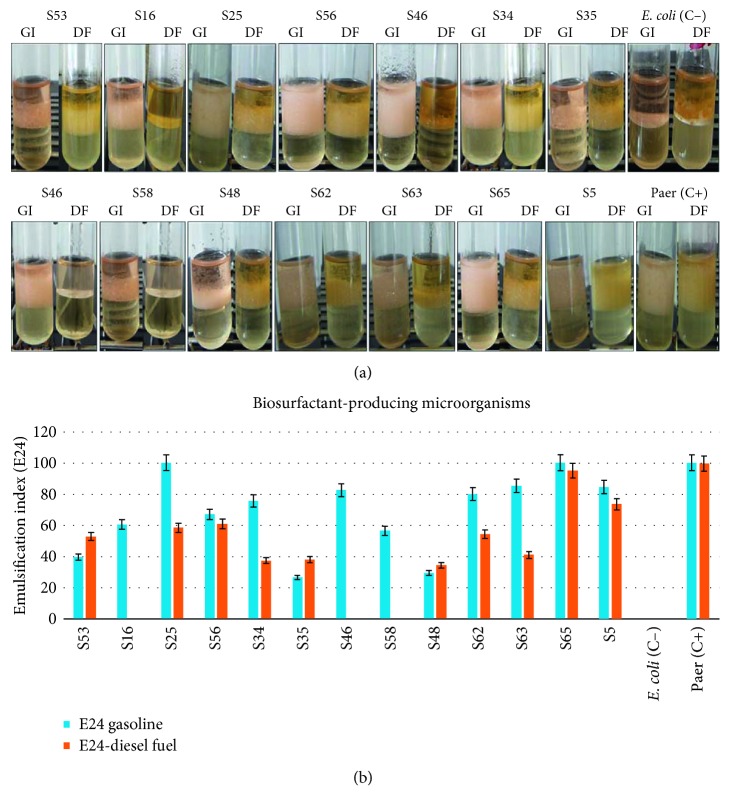
(a) Evaluation of emulsion index after 24 hours (E24) of randomly chosen strains in the presence of gasoline or diesel fuel hydrocarbons. Gl: gasoline; DF: diesel fuel. S53: *Staphylococcus* sp. strain S53; S16: *S. aureus* strain 16; S25: *S. aureus* strain 25; S56: *Bacillus cereus* strain 56; S34: *B. cereus* strain 34; S35: *B. cereus* strain 35; S46: *B. sp. strain* 46; S58: *B. sp. strain* 58; S48: *B. subtilis* 48; S62: *B. licheniformis* strain 62; S63: *B. amyloliquefaciens* strain 63; S65: *P. aeruginosa* strain 65; S5: *A. nosocomialis* strain 5; *E. coli* (C−): lab negative control; Paer (C+): *Pseudomonas aeruginosa* positive control. (b) Percentages of biosurfactant-producing bacteria in the presence of gasoline and diesel fuel hydrocarbon for different bacterial strains.

To highlight how bacteria can grow in wastewater with more successful viability, we assessed the production of biosurfactants by conducting a qualitative test called emulsion index (E24), from inoculated precultures in flasks containing the nutrient broth. Incubation has been done overnight at 37°C. As results illustrated in Figure 3(a), we showed that among the 34 bacterial strains selected according to the profile of degradation and/or tolerance to hydrocarbons (gasoline and/or gas oil), 41.17 % (14) strains were biosurfactant-producing including *Bacillus* sp., *Staphylococcus* spp, and *P. aeruginosa*. Most strains produce biosurfactants with an E24 greater than 50%. *S. aureus* (S25) emulsified 100% gasoline and 58.51% diesel fuel. The strain of *A. nosocomialis* (S5) produces 84.6% Gl and 73.53% DF. Both strains of *Bacillus cereus* (S34 and S35) emulsify also on gasoline as on diesel fuel in the range of 20 to 75%. *Bacillus* sp (S46) was also better by emulsifying about 82% (Figure 2(b)). *B. amyloliquefaciens* strain 63 has E24 ranging to 80.03 % Gl and 54.33 DF and *B. licheniformis* strain 62 85.15 Gl and 40.97 DF (Figure 3(b)).

### 3.4. Effect of Consortium in the Degradation of Hydrocarbons and the Secretion of Biosurfactant

**Figure 4:**
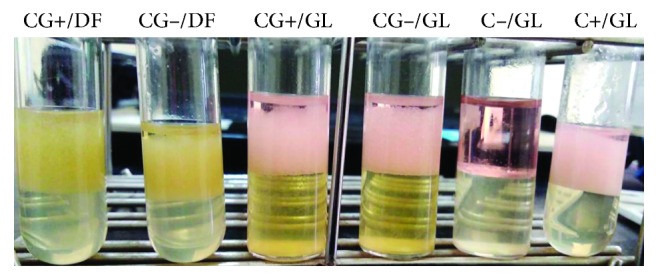
Emulsion index (E24) of consortium of each bacteria group: DF: diesel fuel, GL: gasoline, (C−): negative control including *E. coli* (lab strain), (C+): positive control including *P. aeruginosa* (lab strain), (CG+): consortium of Gram-positive bacteria including *Staphylococcus* spp. S53, *S. aureus* S16, *S. aureus* S25, *S. aureus* S56, *Bacillus cereus* S34, *B. cereus* S35, *B*. sp. S46, *B*. sp. S58, *B. subtilis* S48, *B. licheniformis* S62, and *B. amyloliquefaciens* S63. (CG−): consortium of Gram-negative bacteria including *A. nosocomialis* S5, *A. haemolyticus* S38, and *P. aeruginosa* S65. B: Emulsion index (E24) in the presence of gasoline and diesel fuel for different bacterial strains.

To better understand the cell-cell interaction roles in guppy divergence and adaptation, Gram-positive and Gram-negative bacteria were pooled together. We first assessed for consortium to degrade and/or tolerate hydrocarbons by using the BH medium supplemented with hydrocarbons from gasoline or diesel fuel. Growth was done after 2 and 4 days for the group of Gram-negative bacteria and 1 and 3 days for the Gram-positive bacteria group (data not shown). In addition, the evaluation of E24 also found that each group is able to secrete biosurfactant ranging between 97 and 100% (Figure 4).

### 3.5. Determination of Minimal Inhibitory Concentration (MIC)

**Figure 5:**
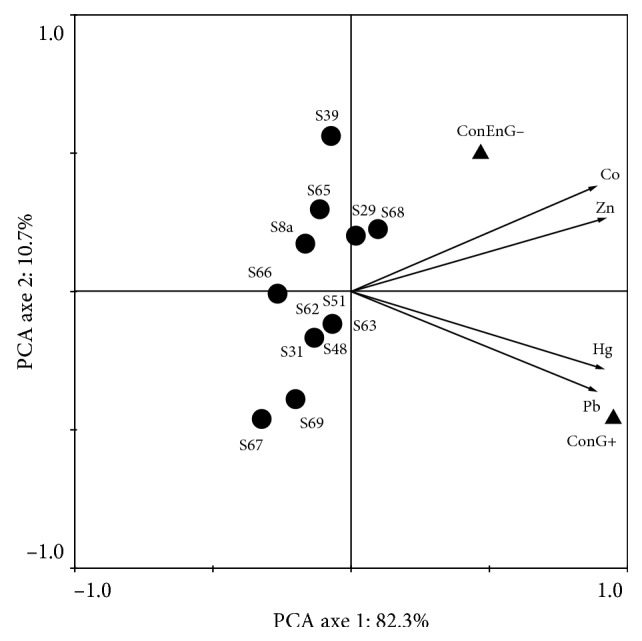
PCA of effect on minimal inhibitory concentration (MIC) on bacteria and consortia. PCA: principal component analysis. S69: *S. flexneri* strain 69, S48: *B. subtilis* strain 48, S63: *B. amyloliquefasciens* strain 63, S62: *B. licheniformis* strain 62, S51: *B. sp. strain* 51, S68: *Enterobacter* sp. strain 68, S39: *E. coli* strain 39, S8a: *S. flexneri* (S31), *Salmonella* spp. (S29), *Salmonella enterica* ssp*.arizonae* (S66), *P. aeruginosa* (S65), *Citrobacter* spp. (S67), *E. coli* strain 8a, CongEnG−: consortium of Gram-negative bacteria group, and ConG+: consortium Gram-positive bacteria group.

We first evaluated the presence of trace metals, metalloids, and halogens in wastewater by finding different concentrations of Pb, Zn, Cu, Fe, Al, Mg, and Cl (Table 2) in the sites of sampling. Trace heavy metals have been identified in the four sites of sampling including Pb, Zn, Cu, and Mg, and we showed that bacteria were able to grow in low concentration, 0.5‰ each. Then, we calculated the MIC of random chosen bacteria in Hg, Co, Zn, and Pb. Data showed that for Hg, the MIC was about 2 and 4‰, Co (2 and 5‰), Zn (9 and 18‰), and Pb (22 and 28‰). Surprisingly, the MIC of the consortium of enterobacterium and Gram-positive bacteria were higher (Figure 5). The consortium Entero G includes Hg (7‰), Co (8‰), Zn (30‰), and Pb (30‰). The consortium seems to be the most interesting one (Figure 4). The consortium of Gram-negative bacteria includes Hg (12‰), Co (11‰), Zn (40‰), and Pb (45‰). The consortium was more shifted to resist to high concentration. So the consortium of Gram-negative bacteria was close to Coand Zn, and the consortium of Gram-positive bacteria was close to Hg and Pb. This shows that bacteria should work together to coordinate specific tasks such as cell-cell interaction.

### 3.6. Effect of Metals on Enzymatic Activity

**Figure 6:**
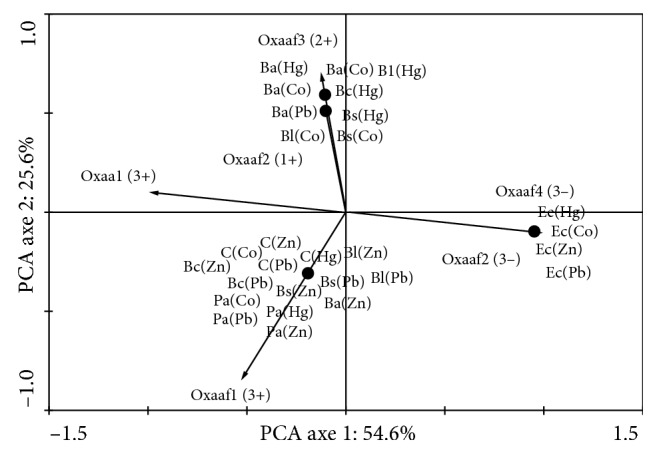
PCA of cytochrome oxidase enzymatic activity. Hg: mercury, Co: cobalt, Pb: plomb, and Zn: zinc. 1+: weak activity; 2+: moderate activity; 2+: excellent growth; 3−: no activity. Oxaa1 (3+): cytochrome oxidase activities in the absence of trace metal with excellent activity. Oxaa2 (3−): Cytochrome oxidase activities in the absence of trace metal with no activity. Oxaaf1 (3+): cytochrome oxidase activities after trace metal incubation with excellent activity. Oxaaf2 (1+): cytochrome oxidase activities after trace metal incubation with weak activity. Oxaaf3 (2+): cytochrome oxidase activities after trace metal incubation with moderate activity. Oxaaaf4 (3−): cytochrome oxidase activities after trace metal incubation with no activity used as negative control. Bs: *Bacillus subtilis* strain 48, Ba: *B. amyloliquefaciens* strain 55, Bl: *B. licheniformis* strain 62, Bc: *B. cereus* strain 35, C: consortium including S35, S48 S62, and S63, Ec. *E. coli* lab strain used as negative control, and Pa: *Pseudomonas aeruginosa* lab strain used as positive control.

Oxidase-positive strains such as *B. cereus* S35, *B. subtilis* 48*, B. licheniformis* 62, and *B. amyloliquefaciens* S63 and consortium including S35, S48, S62, and S63. Lab strains such as *E. coli* and *P. aeruginosa* have been used as negative and positive controls. Bacteria were grown under the MIC previously assessed. After incubation with trace metal, no chosen strains were affected on cytochrome oxidase activity according to Figure 5. As a result, the consortium seems to be the most interesting one with excellent activity (Figure 6).

## 4. Discussion

In this study, we estimate aerobic heterotrophs in the intestine of gut tract which is about 2 × 10^5^ CFU/g. It has been reported that dense bacterial populations occurring in the digestive tract are in the range of 10^8^ UFC heterotrophs/g and ∼10^5^ UFC anaerobes/g [24–27]. There are numerous variations between different species of fish based on the morphology of the gastrointestinal tract. The minimal differences between these studies could be explained by the type of food and the environment in which the fish live [7, 28, 29].

Characterization of the digestive microbiota of *P. reticulata* by standard microbiological methods yielded 72 isolates. The identification of the strains by the molecular biology technique allowed deepening the knowledge on the guppy microbiota digestive. Microbial communities have been consisted of genetically and ecologically distinct consortia. In order to establish a correlation between the gut microbiota and *P. reticulata*, we first identified microorganisms by using biochemical and molecular techniques. The culturable bacteria found in the guppy digestive tract are represented by: *E. coli*, *Shigella* spp., *S. flexneri*, *Salmonella* spp., *E. agglomerans*, *Salmonella enterica* ssp*.arizonae*., *Pseudomonas* spp., *P. aeruginosa, Klebsiella* spp., *K. pneumoniae*, *A. nosocomialis*, *A. haemolyticus*, *Staphylococcus* spp., *S. aureus*, *Bacillus* spp. including *B. subtilis*, *B. amyloliquefaciens*, *B. licheniformis*, *B. altitudinis, B. anthracis, B. marisflavi, B. megaterium*, and *B. cereus*. A wide range of taxa has been previously associated with the digestive tract of adult freshwater fish [30, 31]. Yeasts were not found in this study. Previous studies have showed that fish microbiota is close to same genera identified in this work [10, 25, 32–34]. Microbiota of the gut fish system appears to vary with the complexity of the fish digestive system [35]. Some assessments into the biodiversity of bacterial flora of the gastrointestinal tract of fish have shown that facultative Gram-negative and anaerobic bacteria such as *Bacillus* spp. to be part of the endogenous florae of freshwater fish. Such species are not dominant but they can regularly colonize the digestive tract of fish [8].

Hansen and Olafsen demonstrate that bacteria present in water are able to infiltrate the gastrointestinal tract of fish larvae even before the first feeding [36]. Using Enterosystem 18R combined with molecular techniques, the same genera we found in the intestine of guppy fish have been isolated from wastewater. This includes *Salmonella* spp*., Shigella* spp*., Escherichia* spp*., Bacillus* spp*., Pseudomonas, Citrobacter* spp., and *Salmonella enterica* ssp*. arizonae.* The bacterial diversity of the digestive tract studied shows that the bacteria of the genus isolated from water were found in the digestive tract of the fish. Fish are extremely dependent on their environment; the microorganisms present in the environment can colonize the digestive system by several routes such as the food route, the respiratory tract via the gills, the cutaneous pathway, and the maternal route by transfer to eggs. This colonization would follow the establishment of symbiotic mechanisms related to the use of food for better adaptability in the *P. reticulata* ecosystem.

One provocative investigation of this study has been consisted to assess the ability of bacteria isolated from the digestive track of guppy fish to biodegrade gasoline and diesel fuel hydrocarbons. We showed that among 72 isolates, 36.1% are able to degrade and/or tolerate gasoline hydrocarbons. For diesel fuel criteria, 29.1% are able to degrade and/or to tolerate diesel fuel hydrocarbons. We also showed that the consortium of different groups of Gram-staining bacteria can degrade and/or tolerate gasoline and diesel fuel hydrocarbons more after 2 days only. A couple of strains including *Bacillus* spp, *Pseudomonas* spp., *Staphylococcus* spp., *Shigella* spp., *E. coli*, *Salmonella* spp., and *Acinetobacter* spp. have been identified to be able to grow on BH agar supplemented with different gasoline and diesel fuel hydrocarbons. The complex chemical nature of the hydrocarbons present in diesel fuel could be easily degraded as shown [37–39].

In addition, we have showed that among isolates, 41.17% strains are biosurfactant-producing. A special attention was carried out with *Bacillus* spp and *Staphylococcus* spp., and *Pseudomonas* spp and *Acinetobacter* spp. having a better E24 of hydrocarbons varying between 75% and 100%, respectively. In this work, we also showed that the E24 of enterobacterium consortium was about 99 %. This consortium includes Gram-negative bacteria such as *Pseudomonas* spp and *Acinetobacter* spp. The consortium of Gram-positive bacteria including *Bacillus* spp. and *Staphilococcus* spp. also showed E24 about 98%. Different bacteria species could easily secrete at the same moment a landscape of biosurfactant encompassing rhamnolipid, surfactin, and lipopeptides [40–43] by empowering the guppy adaptability. Alone or together with consortium, bacteria strains could protect the guppy fish in the polluted environments by producing biosurfactants in the digestive tract once the organic pollutants get in touch with the digestive tissues. Recently, it has been confirmed that bacterial strains of the genera of *Staphylococcus* spp. [44] and *Bacillus* spp. [45] play an important role in bioremediation by degrading the hydrocarbons present in the polluted areas and using them as the only carbon source [46]. These bacteria are able to produce biosurfactants with E24 up to 50%. Our results are particularly interesting regarding the ability of isolated strains to emulsify hydrocarbons. This could highlight to be a clear mechanism showing relationship between emulsion activity, cell adhesion to hydrocarbon, and growth rate of isolates on gasoline and/or gas oil.

It has been previously demonstrated the capacity of bacteria for surviving in toxic heavy-metal concentrations [14, 18, 19]. Here, we have shown that microbiota landscape isolated from gut systems can tolerate Hg, Co, Zn, and Pb. The MIC showed mercury (Hg) between 2 and 4‰, cobalt (Co) 2 and 5‰, zinc (Zn) 9 and 18‰, and plomb (Pb) 22 and 27‰. Zn and Pb were the trace metal for which the rate of tolerance was significantly higher. In addition, the consortium value of trace metal including Hg, Co, Zn, and Pb was higher compared with the strain tested alone. This clearly reinforces the fact that the microbiota landscape confers to guppy fish the ability to endure the selective pressures of their environment. Trace metal and hydrocarbon are present in many ecosystems. Associations between trace metals and guppy, *P. reticulata* have been previously illustrated. The effects of some trace heavy metals have also shown that *P. reticulata* is able to adapt the ecosystem contaminated with trace metals. This finding is the first one to demonstrate the direct involvement of landscape microbiota in the digestive track of guppy fish [16].

We also showed that Hg, Co, Zn, and Pb had no visible effect on cytochrome c oxidase activity on *B. cereus* (S35), *B. subtilis* (S48), *B. amyloliquefaciens* (S55), and *B. licheniformis* (S62) growth. However, it is noteworthy that bacteria could also utilize an alternative enzyme for hydrocarbon degradation. Investigation in this way could be interesting. The cytochrome activity of consortia including S35, S48, S62, and S55 has not been affected. This is highlighted to understand that bacteria communities should work together to achieve their best. These findings seem to be the first one by demonstrating the direct involvement of microbiota landscape in the digestive track of guppy fish by highlighting that together with different microbiota genera, guppies are able to adapt in wastewater contaminated with hydrocarbon and trace metals. It is also unwise and difficult to give this entire role to microbiota for the mechanisms of *P. reticulata* adaptation in oil-contaminated waters including cell-cell interaction. Additional physiological factors may influence the adaptation of guppy fish. Response results in morphological, physiological, and even genetic differentiation, paralleling with microbiota growth [47]. In addition, the bacterial enzymes secretion machinery could increase the systematic degradation of gasoline and/or diesel fuel hydrocarbons or the undesirable products. Future studies should also deeply assess relationship between microbiota and *P. reticulata* based on the physiology aspects.

## 5. Conclusion

Our findings tried to illustrate adaptive mechanism abilities among guppies in Brazzaville wastewaters. Evolution has allowed these hydrocarbon- and heavy-metal-adapted microorganisms not to simply survive, but also to grow successfully under the extreme conditions of hydrocarbon habitats, through a variety of microbial aspects and physiological adjustments in their genomes. Within *P. reticulata* is given the opportunity to explore the microbiological related to biological strategies to adapt in vivo coping with high hydrocarbon concentration and heavy metal. Guppies have developed networks of adaptation mechanisms to protect against wild range of pollutants including hydrocarbons and trace heavy metals. Cell-cell interaction could be the most attractive way to keep on investigating by seeing different molecules involved in the adaptation.

## Figures and Tables

**Table 1:** Isolates identified in this work.

Number of isolates	Identification method	Strains
**48**	(i) Sporulation test (ii) Mossel medium (iii) 3% KOH	*Bacillus cereus* (S2, S3, S4, S6, S7, S9, S10, S12, S13, S17, S18, S20, S21, S24, S28, S20, S56, S57, S58, S59) *Bacillus* spp. (S1, S11, S19, S22, S23, S27 S32, S43, S44, S47, S50, S51)
	(i) Sporulation test (ii) Mossel medium (iii) 3% KOH (iv) 16S rRNA gene	*Bacillus cereus* (S34, S35, S64) *Bacillus subtilis* (S48) *Bacillus amyloliquefaciens* (S63) *Bacillus licheniformis* (S62) *Bacillus altitudinis* (S42, S15) *Bacillus megaterium* (S14, S32, S52, S45, S46) *Bacillus marisflavi* (S54, S55, S60) *Bacillus anthracis* (S61)

**6**	(i) Chapman (ii) Medium (iii) 3% KOH (iv) 16S rRNA gene	*Staphylococcus* spp. (S41b, S53) *S. aureus* (S16, S25, S30, S53)

**16**	(i) EMB (ii) SS (iii) 3% KOH (iv) Enterosystem 18R (v) Targeted genes: *icsB* (*Shigella* spp.) and *invG* (*Salmonella* spp.)	*Pseudomonas aeruginosa* (S65) *Pseudomonas* spp. (S70) *Salmonella enterica* ssp*.arizonae.* (S66) *Citrobacter* sp.(S67) *Enterobacter* sp. (S68) *Escherichia coli* (S8a, S39) *Shigella flexneri* (S8b, S31, S69) *Shigella* sp. (S52) *Salmonella* sp. (S29, S33) *Klebsiella pneumoniae* (S26) *Klebsiella* spp. (S37) *Enterobacter agglomerans* (S36)

**2**	(i) EMB (ii) 3% KOH (iii) 16S rRNA gene	*Acinetobacter nosocomialis* (S5) *Acinetobacter haemolyticus* (S38)

**Table 2:** Evaluation of heavy trace metals in the site of sampling.

Wastewater sampling	Pb (mg/l)	Zn (mg/l)	Cu (mg/l)	Cl (mg/l)	Fe (mg/l)	Al (mg/l)	Mg (mg/l)
Site 1	1.19	0.07	0.57	19.2	>1	<0.01	6
Site 2	0.43	0.11	<0.5	2.4	0.24	<0.01	10
Site 3	0.34	0.04	<0.5	1.7	<0.1	<0.01	<5
Site 4	0.45	0.02	<0.5	>25	<0.1	<0.01	0

## Data Availability

The Excel sheet including the data used to support the findings of this study is available from the corresponding author upon request.
